# The effectiveness of EMR implementation regarding reducing documentation errors and waiting time for patients in outpatient clinics: a systematic review

**DOI:** 10.12688/f1000research.45039.2

**Published:** 2021-10-11

**Authors:** Salem Albagmi

**Affiliations:** 1 Department of Health Information Management, Prince Sultan Military College of Health Sciences, P.O. Box 33048, Dammam, 31448, Saudi Arabia

**Keywords:** EMR, medical documentation, documentation errors

## Abstract

**Background: **Electronic medical records (EMRs) refer to the digital copies of paper notes prepared in the physician’s office, outpatient clinics and other departments in health care institutes. EMRs are considered to be significant and preferable to paper records because they allow providers to keep accurate track of patient data and monitoring over time, thus reducing errors, and enhance overall health care quality. The aim of this systematic review was to highlight the significance of EMRs and the effectiveness of implementation regarding reducing documentation errors and waiting time for patients in outpatient clinics.

**Methods: **PubMed, Central, Ovid, Scopus, Science Direct, Elsevier, Cochrane , WHO website and the McMaster University Health Evidence website from 2005-2020 were searched to identify studies that investigated the association between the EMR implementation and documentation error and waiting time for patients. A reviewer screened identified citations and extracted data according to the PRISMA guidelines and data was synthesized in a narrative manner.

**Results: **After full text examination of the articles selected for this literature review, the major themes of relevance that were identified in the context of reducing documentation errors and waiting time for patients in outpatient clinic include: reduction of medical errors because of fewer documentation errors resulting from EMR implementation and reduction of waiting time for patients due to overall improvement of system workflow after use of EMRs.

**Conclusion: **In summary of the reviewed evidence from published material, the implementation of an EMR system in any outpatient setting appears to reduce documentation errors (medication dose errors, issues of prescription errors). It was also seen that in many settings, waiting time for patients in outpatient clinics was reduced with EMR use, while in other settings it was not possible to determine if any significant improvement was seen in this aspect after EMR implementation.

## Introduction

The general definition of electronic medical record (EMR) systems is an electronic record of health-associated data of a person which can be made, collected, organized and consulted by authorized doctors as well as health care staff in a health care institution. EMRs usually refer to digital copies of the paper notes made in the doctor’s office, outpatient clinics and other health care institutions ( Hyman *et al.*, 2012). EMRs consist of the doctor’s official notes and other data or information documented by and for the doctors within that office, clinic, or health care institution ( Manca, 2015). EMRs are generally utilized by the providers for diagnostic purposes and treatment (George and Bernstein, 2009). EMRs are far more important than real paper records since they allow the providers to keep accurate track of patient data over time, help in identifying patients who have to be scheduled for preventive care or vital screenings, allow consistent monitoring of patients, and enhance the overall provided health care quality ( DesRoches *et al.*, 2008; Donsa *et al.*, 2016; Cloete, 2015).

The EMR system once implemented has the acumen to give significant advantages to doctors and outpatient clinical health centers as well as the patients and the overall system of health care institutes ( Agrawal and Wu, 2009). The EMR system has been shown to smooth-out the workflow pathways while enhancing the overall quality of patient care as well as patient safety ( DesRoches *et al.*, 2008; Franklin and Puaar, 2019). The benefits provided by the implementation of an EMR system in a health care facility are many and include the following:

1) Doctors and other health care staff like the nursing staff have quick and instant access to patient medical data or records like previous diagnoses, known drug reactions as well as allergies, laboratory results, and currently prescribed drugs ( DesRoches *et al.*, 2008; Amato *et al.*, 2017).
2) Many electronic medical record systems allow physicians to block special notes or parts of records from the patient portal. This can be helpful in rare cases where the doctor is sure that reading notes can harm the patient. However, with very few exceptions, HIPAA grants patients the right to access prescriptions. Therefore, physicians who refuse to review notes online should discuss this with patients in advance.
3) It is possible for the outpatient clinic staff to gain access to new and past test results from other departments or even other providers in clinics that use multiple care services ( Manca, 2015).
4) Computerized provider order entry (CPOE) is an easier and quicker way of entering and sending medical, laboratorial, radiological and pharmacy prescriptions than paper documents, thus reducing the probability of errors.
5) EMRs are linked to provision of computerized decision-support systems which are accurate in preventing adverse drug interactions and enhancing the overall use of best clinical practices in outpatient settings ( Kavanagh, 2017).
6) Use of EMRs ensures that there is safe, private electronic communication between the health care providers and patients ( Kavanagh, 2017).
7) The patient is also able to access their personal health records, disease management strategies as well as other needed health-related information sources which was not always possible with paper records ( Noraziani *et al.*, 2013).
8) Implementation of an EMR system also computerizes the patient administration systems like their scheduling protocols ( Noraziani *et al.*, 2013).
9) EMRs provide standardized and streamlined methods of storing electronic data and allow timely reporting that in turn improves patient safety as well as disease surveillance programs ( Radley *et al.*, 2013; Noraziani *et al.*, 2013).

Medication errors and increased waiting time for patients due to manual documentation on paper have been found to be significantly associated with compromised health care quality ( Keers *et al.*, 2013). There should be a system that can be used to minimize errors and improve the quality of life of patients. Literature is available discussing the contribution of EMRs and they have been found to improve the health care quality ( Wali *et al.*, 2020; Lin *et al.*, 2020). However, limited studies are available discussing the variables that we have considered in our study as the outcome variable. Therefore, we conducted this systematic review to particularly focus on the reduction in documentation errors and patient waiting time in the outpatient clinic setting by the implementation of EMRs and provide an evidence-based infrastructure to healthcare institutions for better services and facilities.

The duration of task completed at EHR-based facilities can be contributed through the duration of users experience with EHR. Studies have mentioned that user familiarity with a system is associated to the amount of time per task. Most of the studies have examined reduced productivity in hospitals shortly after implementing EHR. The reduced productivity usually enhances as users become more familiar with the new system and establish the relevant abilities for using the system efficiently. In some cases, extensive time duration is needed for performing tasks may continue, which can be demonstrated by an EHR system having additional functions and being more complicated as compared to a relatable paper-based system. The additional characteristics and functions could result in a longer run for completing tasks. It is hypothesized that the patients spend at the EHR-based centers will reduce with time after implementation.

## Methods

### Reporting

The Preferred Reporting Items for Systematic reviews and Meta-Analyses (PRISMA) guidelines have been used in this literature review since this is an already validated methodology for choosing the final articles to be used from the results obtained within published literature relevant to this research topic (Albagmi, 2020). The PRISMA guidelines, as well as being the format for conducting and reporting systematic literature reviews, were selected due to the fact that the overall conceptual ideas and the main aims of the research topic of this project are well suited to the PRISMA guidelines. In addition, in order to carry out an accurate or valid literature review for the purposes of summarizing the various aspects of a chosen public health topic, reviews have been documented as being of more relevance when using already validated methodologies like the PRISMA guidelines ( Moher *et al.*, 2009; Page *et al.*, 2021).

### Search strategy and eligibility criteria

The search strategy was performed in a manner that the methodology can be repeated and employed (see Box 1). A systematic search of the literature published between 2005 and 2020 was performed on PubMed, Central, Ovid, Scopus, Science Direct, Elsevier, Cochrane using free keywords. The reference lists of relevant articles (including primary and secondary results), and grey literature (including PROSPERO and reports of relevant stakeholder organizations - WHO website and the McMaster University Health Evidence website) were screened. The search for relevant articles to be included in the literature review was done after first choosing the appropriate key words and key concepts pertaining to the main research goals of this literature review.

Box 1. **Search strategy (PubMed).**

Search: **efficiency or impact which EMR implementation, outpatient clinics**

"efficiences"[All Fields] OR "efficiency"[MeSH Terms] OR "efficiency"[All Fields] OR "efficiencies"[All Fields] OR "efficient"[All Fields] OR "efficiently"[All Fields] OR "efficients"[All Fields] OR (("impact"[All Fields] OR "impactful"[All Fields] OR "impacting"[All Fields] OR "impacts"[All Fields] OR "tooth, impacted"[MeSH Terms] OR ("tooth"[All Fields] AND "impacted"[All Fields]) OR "impacted tooth"[All Fields] OR "impacted"[All Fields]) AND ("empir musicol rev"[Journal] OR "emr"[All Fields]) AND ("implementability"[All Fields] OR "implementable"[All Fields] OR "implementation"[All Fields] OR "implementation s"[All Fields] OR "implementational"[All Fields] OR "implementations"[All Fields] OR "implementer"[All Fields] OR "implementers"[All Fields] OR "implemention"[All Fields]) AND ("ambulatory care facilities"[MeSH Terms] OR ("ambulatory"[All Fields] AND "care"[All Fields] AND "facilities"[All Fields]) OR "ambulatory care facilities"[All Fields] OR ("outpatient"[All Fields] AND "clinics"[All Fields]) OR "outpatient clinics"[All Fields]))

**Translations**

**efficiency:** "efficiences"[All Fields] OR "efficiency"[MeSH Terms] OR "efficiency"[All Fields] OR "efficiencies"[All Fields] OR "efficient"[All Fields] OR "efficiently"[All Fields] OR "efficients"[All Fields]

**impact:** "impact"[All Fields] OR "impactful"[All Fields] OR "impacting"[All Fields] OR "impacts"[All Fields] OR "tooth, impacted"[MeSH Terms] OR ("tooth"[All Fields] AND "impacted"[All Fields]) OR "impacted tooth"[All Fields] OR "impacted"[All Fields]

**EMR:** "Empir Musicol Rev"[Journal:__jid101513485] OR "emr"[All Fields]

**implementation,:** "implementability"[All Fields] OR "implementable"[All Fields] OR "implementation"[All Fields] OR "implementation's"[All Fields] OR "implementational"[All Fields] OR "implementations"[All Fields] OR "implementer"[All Fields] OR "implementers"[All Fields] OR "implemention"[All Fields]

**outpatient clinics:** "ambulatory care facilities"[MeSH Terms] OR ("ambulatory"[All Fields] AND "care"[All Fields] AND "facilities"[All Fields]) OR "ambulatory care facilities"[All Fields] OR ("outpatient"[All Fields] AND "clinics"[All Fields]) OR "outpatient clinics"[All Fields]

The formulated keywords and concepts were: “efficiency or impact which EMR implementation has had in terms of reducing documentation errors in outpatient clinics” and “efficiency or impact which EMR implementation has had in terms of reducing the waiting time for patients in outpatient clinics.” In accordance with chosen search terms, the study search made use of the keywords as well as the phrases in combination with the Boolean operators “AND” and “OR” in the following manner for search in relevant databases: “efficiency or impact which EMR implementation has had in terms of reducing documentation errors in outpatient clinics” and/or “efficiency or impact which EMR implementation has had in terms of reducing the waiting time for patients in outpatient clinics” ( Cronin, Ryan and Coughlan, 2008; Timmins & McCabe, 2005; Wakefield, 2014).

Each of the chosen databases and websites were used for the search keeping in mind the inclusion and exclusion criteria to find relevant published articles. The inclusion/exclusion criteria used were as follows.

*Inclusion criteria:*

1. All disease and health settings.
2. Articles in the English language.
3. Peer-reviewed articles published 2005-2020.
4. Studies that reported the impact of EMR in reducing documentation error and patient’s waiting time in outpatient clinic settings, either in segregation or as one of the reporting outcomes in a multicomponent studies, were included.

*Exclusion criteria:*

1. Reminder services for appointment and other healthcare activities were not included.
2. Conference proceeding and abstracts were also excluded.

### Study selection and data collection

**Figure 1.**
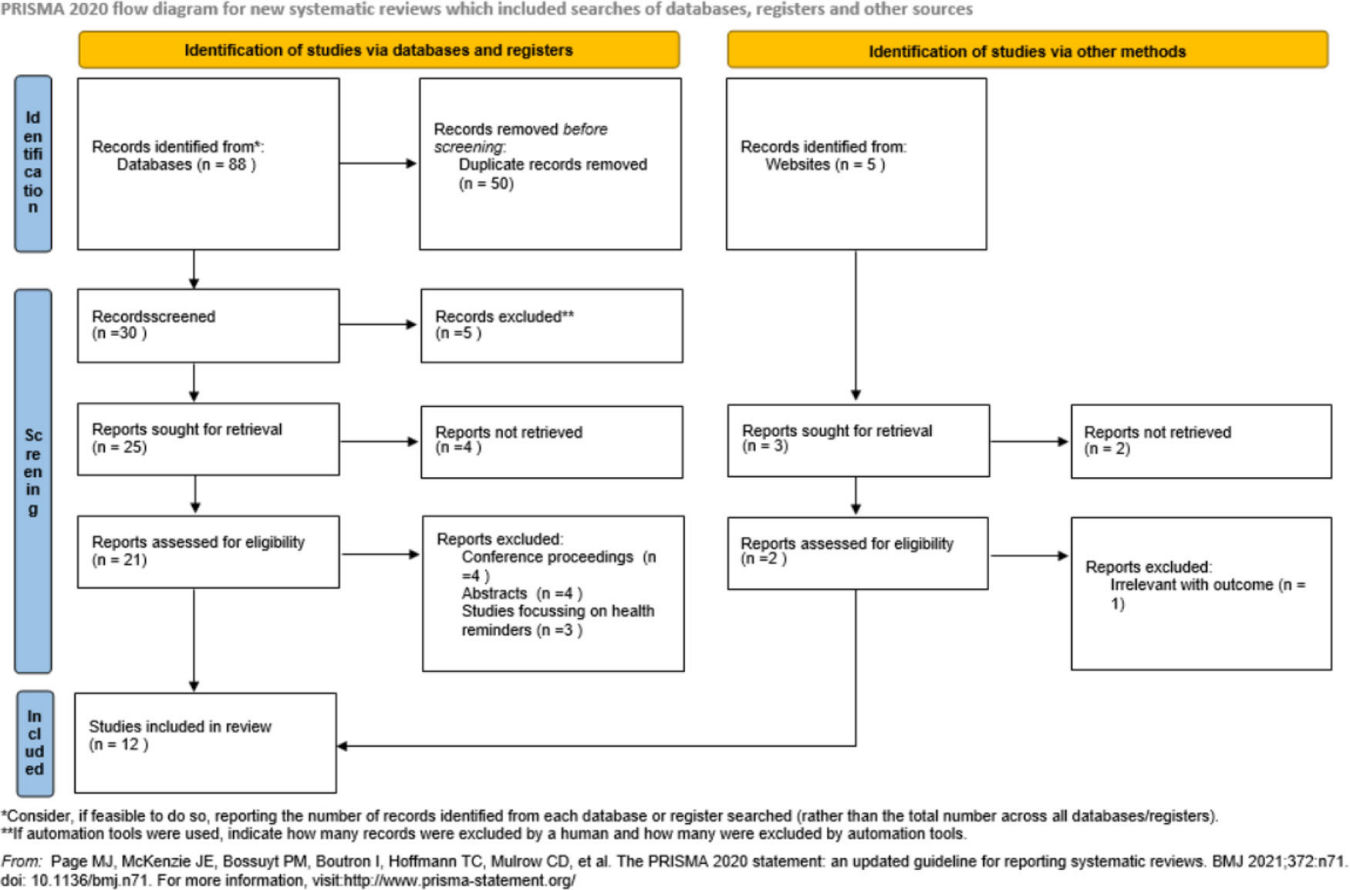
The PRISMA flowchart showing search and selection methodology, PRISMA 2020 flow diagram.

Studies were independently assessed by a single reviewer (SA) and were reported using the PRISMA flow diagram ( Figure 1). Initial screening of studies was based on the information mentioned in the titles and abstracts. Full-paper screening was conducted by the same independent reviewer and extracted in to a standardized structure. A form was created, focused on the detail study characteristics. Primarily, the reviewer collected the data in the form and subsequently it was managed in a master Microsoft Excel sheet (2019) and then tabulated separately for each study. Study investigators were not approached to confirm the data as the included studies were well-defined and outcome was clearly mentioned.

### Data items

The data collected for each study included the name of first author, year of publication, setting characteristics, study duration, participants and, outcomes.

### Primary outcomes

Primary outcomes were any measure related to effectiveness of EMR in:

1. Reducing documentation error,
2. Reducing patient waiting time.

Definitions of variables that were sought out are described in Table 1.

**Table 1.** Definition of variables.

Variable	Definition
Electronic medical records (EMRs)	EMRs usually refer to digital copies of the paper notes made in the doctor’s office, outpatient clinics and other health care institutions
EMR implementation	Use of a electronic software for the documentation by replacing the existing manual documentation method on paper.
Study designs	Type of the study design that was used to conduct the research for example observational studies such as cross sectional, case control studies, cohort studies and interventional studies such as randomized controlled trials. Also the type of reporting the research study that is primary (original articles) and secondary researches (review articles).
Outcome variables	
Impact of EMR on Documentation error	The absence of or decreased in the number of documentation error
Impact of EMR on Patient’s waiting time	Time required to complete the documentation is decreased while patient is waiting. Hence the overall patient’s waiting time is decreased.

### Data analysis and risk of bias assessment

This was a systematic review and hence did not require any statistical analysis. Data was synthesized in a narrative manner. Study quality and bias assessment was done by using a hybrid of the Downs and Black scale and the Newcastle-Ottawa Scale that includes 11 items ( Downs *et al*., 1998; Wells *et al*., 2017) under four themes of reporting, external validity, internal validity for bias and internal validity for confounding to capture the observational and experimental study designs

## Results

### Search results

A search of the above-mentioned chosen databases and sources and the results obtained are shown in Figure 1 (with the use of the PRISMA flowchart for the search strategy for the research question). This literature search made use of many relevant and varied sources so as to obtain the most accurate articles.

After the search was done, a total of 93 articles were found from the databases and other sources. After examining for and removing the duplicates, 30 articles were left. Through the application of the above-mentioned inclusion or exclusion criteria, nine articles were excluded. This resulted in a remainder of 21 articles which were further examined for relevance to the aims of this study’s topic. Following this, nine articles were further excluded for not having relevance in determining the effectiveness of EMR implementation in terms of decreasing or reducing documentation errors and waiting time for patients in outpatient clinics. The remaining 12 articles were utilized for this literature review on the basis of their being relevant to the main aims of this research study (see Figure 1 for the PRISMA flowchart) ( Moher *et al.*, 2009; Page *et al*., 2021).

### Selected study findings and data

The findings and data of the articles chosen for inclusion in this literature review are as shown in Table 2.

**Table 2.** The findings and data of the articles chosen for inclusion in this literature review.

#	First author	Year of publication	Study design	Risk of bias	Outcome
**First group of studies: Reduction of medical errors because of fewer documentation errors resulting from electronic medical record (EMR) implementation**					
1	Priestman, W	2018	Retrospective literature review	Documentation error is studied as one of the variables in this study	312 potential articles were found in initial search, and 117 were used in the review. The findings show that in general, EMR implementation is related to enhancements in documentation and thus leads to decreased prescribing errors. ( Priestman *et al.*, 2018).
2	Manca, P.	2015	Retrospective review	It was a short review and sufficient data was not included.	In summary the findings of this study show that EMRs enhance quality of care, patient outcomes, and safety by reducing documentation errors through better management, reduction in medication errors, reduction in unnecessary testing and better communication between the primary care providers, patients, and other providers linked in care provision. • EMRs have improved efficiencies in workflow and waiting times for patients by decreasing the time needed to pull charts, improved access to full patient data, help in managing prescriptions, improving scheduling of patient appointments, and provision of remote access to patients’ charts. • EMRs recorded point-of-care data which informed and improved practice through quality improvement programs and use of practice-level interventions for reducing documentation error and improving patient safety ( Manca, 2015).
3	DesRoches, C. M	2008	Survey study design	It was survey, may caused response bias and conducted on only ambulatory care setting.	The implementation of EMRs in ambulatory or outpatient clinics by doctors in the USA was found to have a positive effect in reducing documentation errors, whichhelped in improving many aspects of overall provided patient care by enhancing these factors: -Quality of clinical decisions and quality of faster communication with other providers -Quality of communication with patients -Timely access to medical records -Avoiding medication errors in documentation ( DesRoches *et al.*, 2008).
4	George, J	2009	Retrospective literature review	Conducted on only prenatal network.	This study documented that use of EMRs within a prenatal network resulted in a decrease of documentation-related errors and corresponding risks (George and Bernstein, 2009).
5	Agrawal, A	2009	Pilot study design that evaluated the implementation of an EMR system as an intervention	Categorized discrepancy as a severity score according to potential cure and harm was not evaluated.	The findings and evaluation data documented that use of the EMR system coordinated documentation records in a multidisciplinary process based inboth outpatient departments and inpatient wards. This resulted in reduced medication errors on admission. This suggests that EMR systems can be a vital tool in improving patient safety ( Agrawal and Wu, 2009).
6	Radley, D. C	2013	Systematic literature review	It was a review and every study had the different definition of medication error.	The findings of this study showed that implementation of EMR resulted in medication error reduction in many outpatient clinical settings through corresponding reduction of documentation errors in the electronic prescribing done using computerized provider order entry systems ( Radley *et al.*, 2013).
7	Schwartzberg, D	2015	Retrospective literature review	Short period, missing data	The EMR system was implemented with the aim of reducing and eliminating iatrogenic injury caused by avoidable documentation errors in the hospital’s paper-order medication entries. The findings show that the EMRs were useful in reducing errors ( Schwartzberg *et al.*, 2015).
**Second group of studies: Reduction of waiting time for patients due to overall improvement of system workflow after use of EMRs**					
8	Jabour, A. M	2020	Observational study design	Data was not stratified according to patient’s condition and demographics.	The results showed no significant difference in the amount of time spent by patients in the reception area ( *P*=0.26), in the waiting area ( *P*=0.57), consultation time ( *P*=0.08), and at the pharmacy ( *P*=0.28) between the electronic health record and paper based groups. However, there was a significant difference ( *P*<0.001) in the amount of time spent on all tasks between the primary healthcare centres located in metropolitan and rural areas. The longitudinal observation also showed reduction in the registration time (from 5.5 [SD 3.5] min to 0.9 [SD 0.5] min), which could be attributed to the introduction of a Web-based booking system ( Jabour, 2020).
9	AlSarheed, A. H.	2016	Survey study design	Response bias	The study results suggest that software improvements and enhancement of the EMR system in a Saudi hospital’s outpatient clinics reduced the waiting time for their patients ( AlSarheed, 2016).
10	Cho, K. W	2017	Application of theoretical analysis	Only 03 hospitals were examined and waiting time varied with hospital size.	The authors made use of digital data collected from outpatients ‘reception times and consultation finish times’ for calculating the arrival and service rates, respectively (through application of the queuing theory for analysis of outpatients' waiting times). The findings of this study verified that the implementation of EMR contributed to the enhancement of patient services by reducing outpatients' waiting time, or by increasing efficiency ( Cho *et al.*, 2017).
11	Vahdat, V	2018	Quantitative study design utilizing a discrete-event simulation model	Conducted only in dermatology clinic.	The findings suggest that small changes to processes like addition of a few minutes for extra documentation time within the exam room (due to use of EMRs) resulted in significant delays in the timeliness of patient care ( Vahdat *et al.*, 2018).
12	Noraziani, K	2013	Retrospective literature review	Lack of interoperatibility	One of the findings of this review is that use of EMRs in outpatient clinics may save time as it reduces patient waiting times through faster, easier workflow and organizational workflow efficiency ( Noraziani *et al.*, 2013).

### Risk of bias assessment

**Figure 2.**
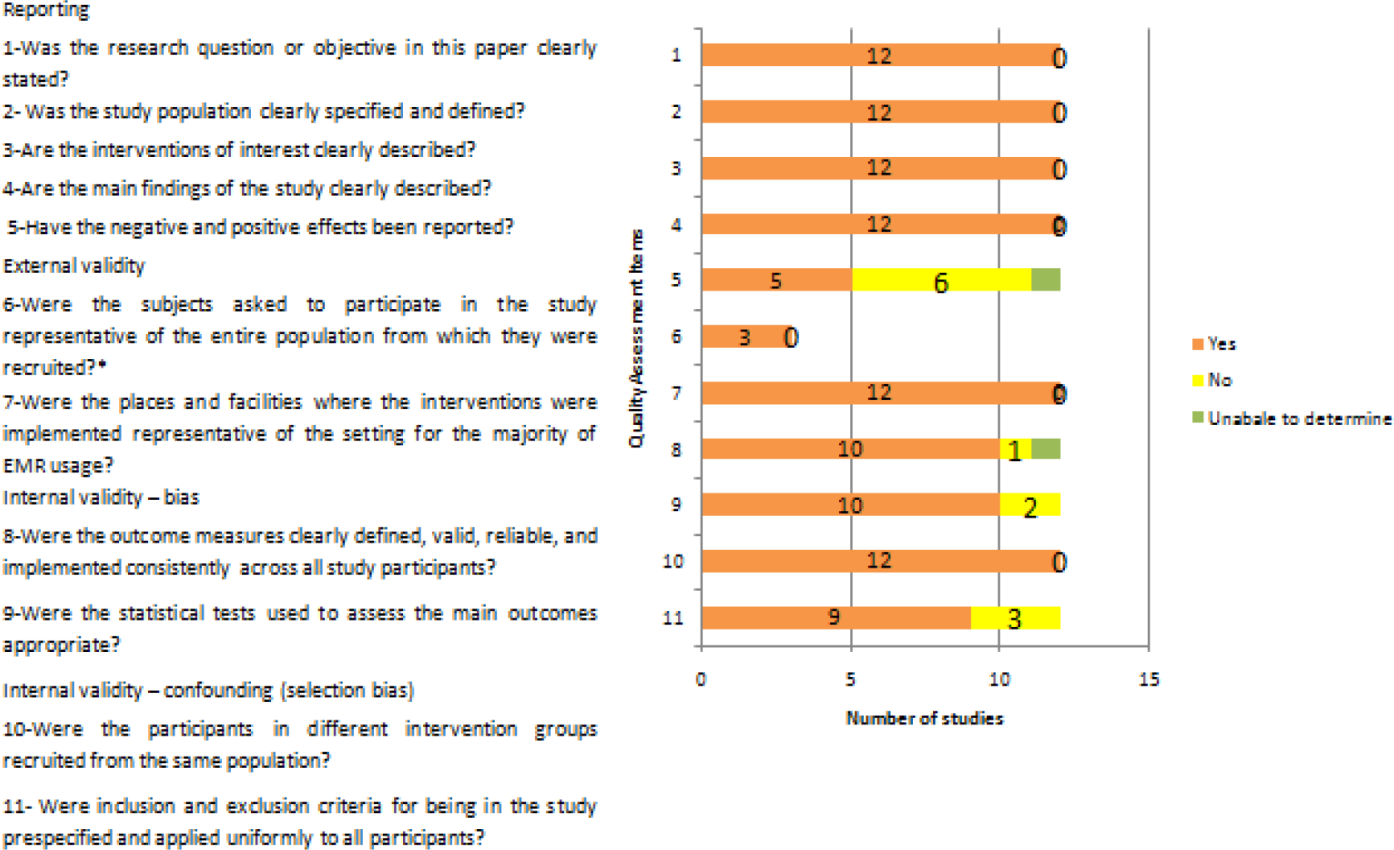
Quality assessment by Downs and Black scale and the Newcastle-Ottawa Scale.

The results of the risk of bias assessment are presented in Figure 2. For question number 6, not all studies recruited participants; out of 12, three studies recruited participants and other studies evaluated databases.

### Key themes identified in the data findings

The review of the selected articles revealed two key themes in the context of how effective the implementation of the EMR system is in reducing documentation errors and waiting time for patients in outpatient clinics, namely: there is a reduction in medical errors because of fewer documentation errors and there is some degree of reduction of waiting time for patients due to overall improvement of system workflow in some settings, after use of EMRs. The two themes tend to overlap and are interlinked. Thus, each aspect has an impact on the other factors and may ultimately improve care quality for patients attending outpatient visits.

## Discussion

The main objectives of this study were to do a literature review to identify the effectiveness of EMR implementation in regard to reducing documentation errors and waiting time for patients in outpatient clinics. The data collected and the emerging themes indicate that there is a decrease in medical errors as a direct result of fewer documentation errors in EMR use ( Priestman *et al.*, 2018; DesRoches *et al.*, 2008; Manca, 2015; Radley *et al.*, 2013) and there is a decrease in waiting time for patients because of overall improvement of system workflow in some settings, after use of EMRs ( Jabour, 2020; AlSarheed, 2016; Cho *et al.*, 2017; Noraziani *et al.*, 2013; Jin, Sivakumar and Lim, 2013), although a contrasting increase was observed in some outpatient studies ( Vahdat *et al.*, 2018).

The implementation of EMRs in outpatient or ambulatory clinics by providers in the US in one study showed a beneficial effect in reducing documentation errors, which in turn enhanced several factors of overall patient care. Improvements were seen in the quality of clinical decisions, quick communication between providers as well as with patients, timely access to all patient medical records in one place and prevention of medication errors in documentation ( DesRoches *et al.*, 2008). Many studies have shown that implementation of an EMR permits doctors to avoid documentation errors since there is improved access to comprehensive patient histories such as clinical data ( Ruano *et al.*, 2016). This in turn reduces waiting time for the patients as doctors spend less time looking for lab reports and other patient data.

The EMR implementation thus provides advantages like fast remote access to patient history, enhanced laboratory result access, medication error alerts, and reminders for scheduling preventive care ( Manca, 2015; Radley *et al.*, 2013). It was also observed that implementation of various forms of the EMR systems can be a vital tool in improving patient safety in outpatient and inpatient settings ( Agrawal and Wu, 2009). As a point of comparison, it was seen that researchers studied and compared the impact of errors in paper-based and computerized EMR diabetes management with decision support in hospitalized patients with type 2 diabetes ( Donsa *et al.*, 2016; Vishwanath, Singh and Winkelstein, 2010). This could be applicable in outpatient clinics too. The results from one study suggest that software improvements and enhancement of the EMR system in a Saudi hospital’s outpatient clinics reduced the waiting time for their patients ( Al Sarheed, 2016).

However, some studies have shown contrasting results to those showing improvements in waiting times of outpatient clinic visitors. A study designed to quantify the downstream effect on patient wait times as well as the total duration of stay because of tiny increments in encounter times resulting from implementation of a new EMR system indicated that use of EMRs added extra documentation time in outpatient clinics and overall led to significant delays in the waiting times for patients ( Vahdat *et al.*, 2018).

Additionally, the type of EHR system used can influence the outcome. The generalizability of finding to PHC centers is unidentified even though the system being used at PHC centers is approved and provided by the government. Moreover, there was limited evidence available for determining the impact of system familiarity on the reception time for the longitudinal part of the study with the instigation of the Web-based booking system after the baseline period.

### Limitations

This review has some limitations. First, due to the strict exclusion and inclusion criteria, the studies that were relevant according to the one of the outcomes but not to the main question were not included. Conference proceedings were excluded that might have contained findings of worth. Second, there was a lack of data within studies regarding setting, type of study and population. In addition, a large number of studies were found on documentation errors as compared to patient waiting time.

## Conclusion

In summary, EMR implementation appears to enhance documentation, screening performance as well as prescription error prevention. The findings presented in this literature review will be of value to outpatient clinics and units that are looking to implement EMR systems. The waiting time for outpatient clinic visits can also be reduced by EMR use as there is implementation of new tools and functionality like the Web-based booking system, which can reduce the waiting time as well as duration of patients’ visits ( Jabour, 2020; Noraziani *et al.*, 2013). However, this needs to be further validated in future studies as some studies actually indicate that implementation of an EMR system led to increased waiting times and delays in care provision in outpatient clinics.

### Future perspective

Although previous versions of EHRs were mainly undertaken as recordkeeping devices, corresponding versions have commenced implementing a “rules engine” that facilitate institutions in developing their own decision-support tools. Therefore, EHR vendors have expanded the functionality of their products, irrespective to integrate Food and Drug Administration approval of their software as a regulated medical device. The difference is essential for two reasons. Firstly, the risks related with health information technology are concerned by the FDA. Secondly, this stresses the burden of developing the decision support tool on the individual institution, which may reduce the likely effect of decision-support, undertaking the amount of time or effort needed for customization. The difference between decision support tools is stressed through alert users toward contradictory or missing documentation versus those that suggested the adjustment of anesthetic depth on the basis of vital signs of patients. Implementing decision support tools is slightly different in anesthesia as compared to most other specialties. Since events are occurred in real-time, and actions might be timely critical, event processing time and network delays must be undertaken for ensuring that alerts are timely and appropriate to the clinician.

## Data availability

### Underlying data

All data underlying the results are available as part of the article and no additional source data are required.

### Reporting guidelines

Figshare: PRISMA checklist for “The effectiveness of EMR implementation regarding reducing documentation errors and waiting time for patients in outpatient clinics: a systematic review” https://doi.org/10.6084/m9.figshare.14791356.v1 ( Albagmi, 2021).

Data are available under the terms of the Creative Commons Zero “No rights reserved” data waiver (CC0 1.0 Public domain dedication).
