# Supplementary material for: The effectiveness of EMR implementation regarding reducing documentation errors and waiting time for patients in outpatient clinics: a systematic review
Source: F1000Res. 2021 Oct 11;10:514. Originally published 2021 Jun 29. [Version 2] doi: 10.12688/f1000research.45039.2 (PMC8738966; doi:10.12688/f1000research.45039.2)
Supplement: Peer review history

# Peer review: Reviewer response for version 2

**DOI:** 10.5256/f1000research.77486.r96603

**Published:** 2022-01-06

**Authors:** Clair M. Sullivan

**Affiliations:** 1 Centre for Health Services Research, Faculty of Medicine, The University of Queensland, Brisbane, QLD, Australia

My comments have been satisfactorily addressed, thank you.

Are the rationale for, and objectives of, the Systematic Review clearly stated?

Partly

Is the statistical analysis and its interpretation appropriate?

Yes

Are sufficient details of the methods and analysis provided to allow replication by others?

Yes

Are the conclusions drawn adequately supported by the results presented in the review?

Partly

Reviewer Expertise:

NA

I confirm that I have read this submission and believe that I have an appropriate level of expertise to confirm that it is of an acceptable scientific standard.

# Peer review: Reviewer response for version 2

**DOI:** 10.5256/f1000research.77486.r96604

**Published:** 2021-10-18

**Authors:** Shankar Srinivasan

**Affiliations:** 1 School of Health Professions, Rutgers University, Newark, NJ, USA

The revised manuscript looks quite apt and responds to the suggestions and recommendations.

Are the rationale for, and objectives of, the Systematic Review clearly stated?

Yes

Is the statistical analysis and its interpretation appropriate?

Yes

Are sufficient details of the methods and analysis provided to allow replication by others?

Yes

Are the conclusions drawn adequately supported by the results presented in the review?

Yes

Reviewer Expertise:

Health Outcomes Research, Health Data Analytics

I confirm that I have read this submission and believe that I have an appropriate level of expertise to confirm that it is of an acceptable scientific standard.

# Peer review: Reviewer response for version 1

**DOI:** 10.5256/f1000research.48111.r89947

**Published:** 2021-08-03

**Authors:** Shankar Srinivasan

**Affiliations:** 1 School of Health Professions, Rutgers University, Newark, NJ, USA

The manuscript describes the systematic review of published literature on the effectiveness of electronic health records in reducing documentation caused errors and also waiting time for ambulatory settings. The duration chosen is quite appropriate (2005 to 2020), given that the widespread implementation (at least in the US) was from 2009 onwards. The author has done due diligence with the selection of the manuscripts for the review by including (and specifying) the appropriate inclusion and exclusion criteria so that another researcher can reproduce the study accordingly. The analyses, results and conclusions drawn are robust and well written in responding specifically to the outcomes in question and also the results for those two outcomes.

 The author, however, hasn't specified or explained why they chose those two outcomes and not others, such as prescription contraindication caused issues, quality of point of care, and so on, which are also included in much literature on EHR/EMR effectiveness.

 Secondly, the manuscript would have been better enhanced with some recommendations for future implementations of EHR/EMR.

 Lastly, is there a difference in the types of ambulatory settings (surgical, physical therapy, urgent care)? These would have enhanced the quality and usefulness of the paper but they do not detract from the overall quality and rigor of the work reported in the existing manuscript and the way it was conducted.

Are the rationale for, and objectives of, the Systematic Review clearly stated?

Yes

Is the statistical analysis and its interpretation appropriate?

Yes

Are sufficient details of the methods and analysis provided to allow replication by others?

Yes

Are the conclusions drawn adequately supported by the results presented in the review?

Yes

Reviewer Expertise:

Health Outcomes Research, Health Data Analytics

I confirm that I have read this submission and believe that I have an appropriate level of expertise to confirm that it is of an acceptable scientific standard.

# Response

**Published:** 2021-09-17

**Authors:** Salem Albagmi

**Affiliations:** Prince Sultan Military College of Health Sciences, Saudi Arabia

**Comment 1:**

 The author, however, hasn't specified or explained why they chose those two outcomes and not others, such as prescription contraindication caused issues, quality of point of care, and so on, which are also included in much literature on EHR/EMR effectiveness.

- **Page No. **5, **Line No.** 97-107, **Actual Content**
- **Removed/Edited/Replaced Content: **Rationale for selecting both variables in the introduction section

**Comment 2:**

 Secondly, the manuscript would have been better enhanced with some recommendations for future implementations of EHR/EMR.

- **Page No. **19, **Line No.** 295-310, **Actual Content**
- **Removed/Edited/Replaced Content: **Future perspective of EHR is added

**Comment 3:**

 Lastly, is there a difference in the types of ambulatory settings (surgical, physical therapy, urgent care)? These would have enhanced the quality and usefulness of the paper but they do not detract from the overall quality and rigor of the work reported in the existing manuscript and the way it was conducted.

- **Page No. **17, **Line No.** 269-274, **Actual Content**
- **Removed/Edited/Replaced Content: **Discussion related to EMR/EHR system is added in the context of types

# Peer review: Reviewer response for version 1

**DOI:** 10.5256/f1000research.48111.r88680

**Published:** 2021-07-19

**Authors:** Clair M. Sullivan

**Affiliations:** 1 Centre for Health Services Research, Faculty of Medicine, The University of Queensland, Brisbane, QLD, Australia

Thank you for this paper.

 A few overarching comments:

1. This paper is very doctor centric. Although it is true that EMRs contain doctor's notes, they actually allow integration of notes from all members of a multidisciplinary team. Please review the paper to include this.
2. The two outcome variables chosen, reduced waiting time and reduced documentation errors, are both important; but I am curious as to why these two were selected out of the 100s of available impacts? Is there a logic behind this? If so, please outline as it appears a little random currently.
3. The terms ambulatory care and outpatients can be confusing. Are you referring to specialised ambulatory care services?  Or primary care? These settings are vastly different and so it is difficult to compare a primary care provider in general practice to a specialised neurosurgery outpatients department.
4. Please review grammar and syntax as this appears a little clumsy at times  e.g avoid calling EMRs magnificent 

Are the rationale for, and objectives of, the Systematic Review clearly stated?

Partly

Is the statistical analysis and its interpretation appropriate?

Yes

Are sufficient details of the methods and analysis provided to allow replication by others?

Yes

Are the conclusions drawn adequately supported by the results presented in the review?

Partly

Reviewer Expertise:

NA

I confirm that I have read this submission and believe that I have an appropriate level of expertise to confirm that it is of an acceptable scientific standard, however I have significant reservations, as outlined above.

# Response

**Published:** 2021-09-17

**Authors:** Salem Albagmi

**Affiliations:** Prince Sultan Military College of Health Sciences, Saudi Arabia

**Comment 1:**

 This paper is very doctor centric. Although it is true that EMRs contain doctor's notes, they actually allow integration of notes from all members of a multidisciplinary team. Please review the paper to include this.

- **Page No. **3, **Line No. **62-66, **Actual Content**
- **Removed/ Edited/ Replaced Content: **The following aspect is added in the introduction section

**Comment 2:**

 The two outcome variables chosen, reduced waiting time and reduced documentation errors, are both important; but I am curious as to why these two were selected out of the 100s of available impacts? Is there a logic behind this? If so, please outline as it appears a little random currently.

- **Page No. **5, **Line No .**97-107, **Actual Content**
- **Removed/ Edited/ Replaced Content: **Rationale for selecting both variables in the introduction section

**Comment 3:**

 The terms ambulatory care and outpatients can be confusing. Are you referring to specialized ambulatory care services?  Or primary care? These settings are vastly different and so it is difficult to compare a primary care provider in general practice to a specialized neurosurgery outpatients department.

- **Removed/ Edited/ Replaced Content: **Not catered - the paper is written and discussed in the context of specialized neurosurgery outpatients department
